# Investigating Spectral Biomarker Candidates for Migratory Potential in Cancer Cells Using Micro-FTIR and O‑PTIR Spectroscopy

**DOI:** 10.1021/acsmeasuresciau.5c00132

**Published:** 2026-01-21

**Authors:** Elisabeth Holub, Nikolaus Hondl, Kai-Lan Lin, Marjaana Parikainen, Cecilia Sahlgren, Bernhard Lendl, Georg Ramer

**Affiliations:** † Institute of Chemical Technologies and Analytics, 27259 TU Wien, 1060, Wien, Austria; ‡ Faculty of Science and Engineering, 1040 Åbo Academi, 20500, Turku, Finland; ¶ InFLAMES Research Flagship, Åbo Akademi University and University of Turku, 20500, Turku, Finland; § Department of Biomedical Engineering, Eindhoven University of Technology, 5631 BN, Eindhoven, The Netherlands; ∥ Institute for Complex Molecular Systems (ICMS), Eindhoven University of Technology, 5612 AJ, Eindhoven, The Netherlands; ⊥ Christian Doppler Laboratory for Advanced Mid-Infrared Laser Spectroscopy in (Bio-)process Analytics, 27259 TU Wien, 1060, Vienna, Austria

**Keywords:** infrared spectroscopy, O-PTIR, FTIR, spectral biomarkers, migratory cells

## Abstract

Routine diagnostic practice for cancer and metastasis relies on a time-consuming staining process and the use of antibodies to detect selected molecular markers and is hence limited by a lack of real-time data and the availability of molecular information. Against this background, techniques based on rapid chemical analysis to identify migratory properties are highly desirable. Fourier-Transform Infrared (FTIR) microspectroscopy has a long history in the label-free identification of infrared marker bands for cancer detection. However, it requires extensive postprocessing of the acquired spectra, is of limited suitability for analysis in aqueous environments, and has poor spatial resolution. To overcome these challenges, we are using a new method termed Optical Photothermal Infrared (O-PTIR) spectroscopy to detect local absorption to establish potential IR tumor markers and classification models. We report on experimental outcomes using machine learning and FTIR microspectroscopy for the classification of cells and the analysis of spectral features reflecting cancer and migratory properties, comparing a commercial FTIR microspectrometer to a custom-built O-PTIR instrument dedicated to spectroscopic measurement and imaging in microfluidic channels.

## Graphical abstract

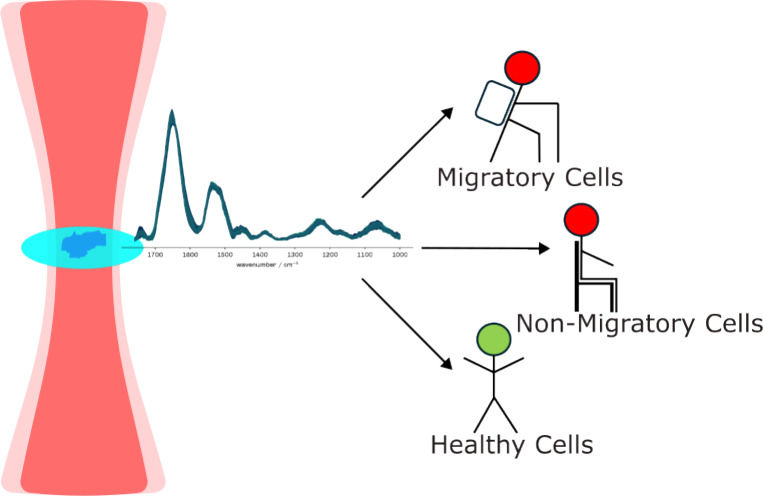

## 1 Introduction

Current diagnosis of cancer and metastasis is based on the qualitative examination of stained tissue sections by a pathologist, which involves a time-consuming staining process and cannot provide real-time information for the intraoperative assessment of tumors.^5,6^ Hence, metastatic sites are detected by imaging tests only after the cancer has spread, while little attempts are made in identifying high-risk patients before a metastatic event.

Moreover, since traditional histology lacks the ability to capture molecular data, treatment decisions are usually guided by the analysis of spatial patterns in stained histological sections, supported by immunohistochemistry, i.e., the use of antibodies to detect the presence and location of certain molecular markers.^7^ However, only a limited number of such markers can be detected simultaneously, making the characterization of a multifactorial disease such as cancer a challenging task.^8^ Dye-free chemical profiling of aggressive and nonaggressive cancer cells and tissues can therefore provide valuable information for risk-adapted monitoring and treatment of cancer.

A noninvasive, label-free and biologically specific method, Fourier-Transform Infrared (FTIR) microspectroscopy has shown great diagnostic potential in disease and cancer detection. The application of the FTIR technique to histological samples gave rise to the term “spectral histopathology” ^9,10^ and includes efforts to distinguish between healthy and tumor cells,^2,4,11,12^ the establishment of cancer markers,^1,13^ and the spectral analysis of several stages of metastasis.^14^

However, the optical diffraction limit restricts the (lateral) spatial resolution of infrared (IR) microscopy to several microns. Furthermore, the strong absorption of infrared radiation by water prevents the routine use of IR spectroscopy in wet tissues or aqueous media. Despite recent technological advances in mid-IR-laser-based microscopes and imaging systems, which allow for significantly faster analysis, IR microscopy is still an absorption-based technique, and measurement in aqueous samples remains challenging.

More recently, a novel method termed Optical Photothermal Infrared (O-PTIR) spectroscopy has broken new ground in overcoming the above-mentioned challenges, achieving submicrometer lateral resolution while at the same time providing the same spectral information as probed in FTIR (micro)­spectroscopy.^15,16^

O-PTIR is a label-free pump–probe technique that combines the biological specificity of IR spectroscopy and the spatial resolution of optical microscopy by using an IR pump and a visible probe. The absorption of the (focused) IR laser induces a local change in the refractive index of the sample. This local refractive-index perturbation acts as a lens and modifies the diameter of the transmitted probe beam. When an aperture is placed before the detector, the power transmitted through the aperture varies with the probe beam diameter, which in turn depends on the strength of the thermal lens and thus the absorbed IR power.

When the IR beam is modulated, the change in detected probe laser power has the same frequency as the pump laser repetition rate and can be extracted from the transmission signal using a lock-in amplifier, referenced to the laser repetition rate. High-frequency modulation of the IR beam and demodulation of the visible probe make it possible to detect minute refractive-index changes against a strong background. The visible laser spot can be focused on a very small area of interest, further reducing the contribution from surrounding material and giving the technique great potential to detect small variations in local chemical structure. With its visible-light lateral resolution, O-PTIR spectroscopy is better suited for studying sample differences at the subcellular level than traditional IR spectroscopic modalities.

Moreover, the use of a visible probe bypasses the strong absorption of infrared radiation by water, allowing infrared spectra to be acquired even in aqueous environments.^17^ This gives the O-PTIR technique a decisive advantage over FTIR microspectroscopy and makes it more suitable for the analysis of hydrated tissues and live cells.

In the present study, our aim was to demonstrate that spectra recorded with our home-built O-PTIR instrument exhibit the same qualitative features as spectra obtained with a commercial FTIR microspectrometer. We then established a classification model to distinguish between aggressive and nonaggressive cancer cells, comparing FTIR and O-PTIR spectra. The primary goal of this work was to verify that migratory cells could be detected in O-PTIR spectra and differentiated from nonmigratory cells using only a limited subset of spectral bands.

A highly aggressive breast cancer cell line was modified to obtain a migratory and a nonmigratory cell type. Two primary endothelial cell lines served as healthy controls. Multiple spectra of all cell samples were collected with a commercial FTIR microspectrometer and a custom confocal O-PTIR instrument (Figure S1). The O-PTIR microscope was designed in transmission mode, making it a versatile tool for measurement in liquid environments and on-chip applications.

Training and test sets were randomly generated for each of the FTIR and O-PTIR data sets for variable selection by the Least Absolute Shrinkage and Selection Operator (LASSO) and classification by Linear Discriminant Analysis (LDA). Past research indicates that supervised machine learning approaches perform better in terms of accuracy when the number of classes is small, and are more robust to imbalanced data.^18^ In this study, there were only 3 cell labels, which were known, making supervised algorithms the natural choice.

LDA is a well-known supervised learning technique that solves multiclass classification problems by modeling the data distribution for each class. The goal of maximizing the separation between different classes is met by finding a linear combination of features that maximizes the ratio of between-class variance to within-class variance.^19–21^ Due to its straightforward concept and wide applicability, LDA has been used for decades and is readily available in software libraries.

A major drawback of LDA is that it cannot be applied when the number of variables is much larger than the number of observations, which is a common problem in spectral analysis. In this case, the within-class covariance matrix of the features is singular, resulting in instability and overfitting.^22,23^ This limitation can be bypassed by reducing the number of input variables via regularization, dimension reduction or variable preselection.^22,24^ Regularization directly modifies the objective function of the classifier,^24^ making it difficult to pinpoint which features are most influential. Instead of directly penalizing the discriminant function, dimension reduction techniques generate new variables from the original ones to preserve as much information as possible.^25^ Again, this approach masks the importance of individual variables and decreases the interpretability of the model. Conversely, variable selection methods identify the most important features while discarding less relevant ones, thus simplifying the model and improving its interpretability.

For this reason, a Least Absolute Shrinkage and Selection Operator (LASSO) was employed prior to classification to select the most relevant features for each data set, reducing the spectral data to only a few wavenumbers. Ridge regression was also tested but resulted in a much larger subset of wavenumbers for the desired classification accuracy, making it difficult to identify distinct spectral marker candidates.

The selected wavenumber subset was subjected to histogram and statistical analysis to understand the importance of the selected wavenumbers for each cell class. Final classification was carried out using a linear discriminant analysis (LDA) model with multifold cross-validation.

The wavenumber ranges output by the LASSO (Tables S2 and S3) correspond to IR cancer markers previously suggested in the literature, such as the symmetric and asymmetric stretching modes of the phosphate bond,^11,26,27^ the Amide I^11^ and Amide II^28,29^ protein bands, CH_2_ bending and asymmetric CH_3_ bending of proteins and lipids,^30^ and the C=O stretching of esterified lipids.^11^

In the present analysis, the phosphate and esterified lipid bands were most strongly related to differences between the migratory and nonmigratory cell groups. The migratory and nonmigratory classes could be distinguished in both data sets even though the class size was much lower for the O-PTIR data. These findings underscore the potential of infrared spectroscopy methods to identify subtle chemical differences in complex biological samples.

## 2 Experimental Section

### 2.1 Instruments and Measurement Parameters

FTIR microspectroscopy was performed with a commercial microscope (Hyperion 3000, Bruker, Billerica, MA, USA). The FTIR microspectrometer is equipped with a nitrogen-cooled focal plane array (FPA) detector for concurrent measurement of 64 × 64 spectra. These hyperspectral images were collected in transmission using a clean part of the substrate as background. Spectra were collected at a spectral resolution of 2 cm^–1^ and a pixel resolution of approximately 2.7 μm within the spectral range of 930 cm^–1^ to 3850 cm^–1^. The pixel resolution was determined from the detector’s field of view (170 × 170 μm^2^) and the number of pixels (64 × 64).

O-PTIR spectroscopy was performed using a custom confocal instrument described elsewhere^31^ (Figure S1). Samples are irradiated with a tunable IR pump laser (MIRcat-QT-z, Daylight Solutions, San Diego, CA, USA) from below; a counter-propagating continuous probe laser (LBX-633, Oxxius, Lannion, France) detects the wavenumber-specific absorption in the sample. The spectral resolution, pulse width and modulation frequency settings of the IR laser were 1 cm^–1^, 500 ns and 50 kHz, respectively. The modulation frequency is a compromise between 1/f noise and the low-pass behavior of the photothermal measurement principle. The frequency of 50 kHz has been found to offer a good compromise. The spectral ranges for each of the four MIRcat-QT-z chips is reported in Table S1. The visible laser power was approximately 1 mW at the sample. In O-PTIR microscopy, lateral spatial resolution is dictated by the visible laser wavelength of 633 nm. For the custom-built instrument reported here, lateral spatial resolution was determined to be below 700 nm.^31^

Both instruments were flushed with dry air to remove atmospheric water vapor and thus avoid misrepresentation of the Amide I and II bands (1600 cm^–1^ to 1720 cm^–1^ and 1500 cm^–1^ to 1560 cm^–1^).^32^

### 2.2 Samples

A highly aggressive, triple-negative breast cancer cell line, MDA-MB-231 WT, was modified using CRISPR-Cas9 to obtain two cell types that would only differ in the migratory properties but were otherwise morphologically identical.^33^ Triple-negative cells test negative for estrogen, progesterone and human epidermal growth factor receptor 2 (HER2) and are characterized by high aggressiveness and low chances of recovery.^34^ A Jagged 1 (JAG1) knockout was performed to create a nonmigratory cell type (MDA-MB 231 KO). Two primary endothelial cell lines (HAoEC, human aortic endothelial cells, and HUVEC, human umbilical vein endothelial cells) were grown for 4 to 6 passages. The measurements of both HAoEC and HUVEC were combined to form a single “Healthy” control group.

There is ample evidence that JAG1 is a ligand for Notch receptors that plays a major role in tumor cell growth, invasion and metastasis.^35–37^ JAG1 suppression reduces migration and invasive capacity in cancer cells.^35–37^ More specifically, JAG1 knock-down has been demonstrated to impair MDA-MB-231 cell motility, while JAG1 overexpression promotes MDA-MB-231 cell proliferation.^37^ Furthermore, JAG1 is strongly expressed by tumor vasculature^38^ and has been associated with vascular invasion, higher tumor grade and poor outcomes in breast cancer patients.^37,39,40^

Cell lines were cultured in their respective optimal growth media. MDA-MB-231 WT and MDA-MB-231 KO cells were maintained in high-glucose Dulbecco’s Modified Eagle Medium (DMEM) supplemented with 10% fetal bovine serum (FBS), 2 mmol L^–1^ glutamine, 100 units/mL penicillin, and 100 μg mL^–1^ streptomycin. HAoEC and HUVEC, along with their specific culture media, were purchased from PromoCell (Heidelberg, Germany). HAoEC were cultured in Endothelial Cell Growth Medium MV with SupplementMix, and HUVEC in Endothelial Cell Growth Medium 2 with SupplementMix. All cell cultures tested negative for mycoplasma and were maintained at 37 °C in a humidified atmosphere containing 5% CO_2_.

For spectral analysis, 50 000 cells per cell line were seeded onto CaF_2_ glass (Crystran Ltd., Poole, UK) of 13 mm diameter and 1 mm thickness and allowed to adhere overnight. The following day, cells were fixed with 4% PFA for 10 min at room temperature, then stored in PBS buffer at 4 °C.

Before analysis, PBS buffer supernatant was decanted and samples were rinsed at least 5 times with distilled water to remove excess PBS and prevent salt crystal formation. The same sample types were used for comparative analysis, but different replicates of the parent cells were used.

### 2.3 Collection and Processing of Spectra

Vibrational spectra are affected by noise and measurement artifacts. Noise sources include environmental fluctuations and instrument noise, while artifacts include optical artifacts, sample impurities, as well as scattering and standing-wave effects.^41^ As these unwanted signal variations can mask spectral features and have a significant impact on the performance of quantitative models,^42^ it is indispensable to remove them from the underlying model data. The preprocessing steps comprise the removal of outliers (e.g., bad pixel correction, removal of spectra with low signal-to-noise ratio (SNR)), baseline correction, smoothing, scattering correction, and normalization.

All postprocessing and data formatting operations were carried out using Python 3, in particular the NumPy^43,44^ and xarray^45^ packages as well as the pybaselines^46^ and scikit-learn^47^ libraries.

#### 2.3.1 FTIR

The FTIR instrument was set to record 64 × 64 points per image; 64 scans were averaged per pixel. In total, spectra from approximately 100 cells per cell class (MDA-MB 231 WT, MDA-MB 231 KO, Healthy) were collected from two different sample batches. The exact class sizes are reported in Table S5. The SNRs at 1650 cm^–1^, calculated per class as the quotient of the mean intensity and the standard deviation, was 50 for MDA-MB 231 WT, 72 for MDA-MB 231 KO, 47 for HUVEC and 51 for HAoEC.

Several postprocessing steps are required to ensure comparability of individual FTIR measurements. The postprocessing workflow is illustrated in Figure 1. First, hyperspectral images (a) were segmented into regions of interest (ROIs, i.e., cells) and low-signal regions, i.e., the background (b). Spectra of low intensity and ROIs containing less than 4 spectra were discarded.

**Figure 1:**
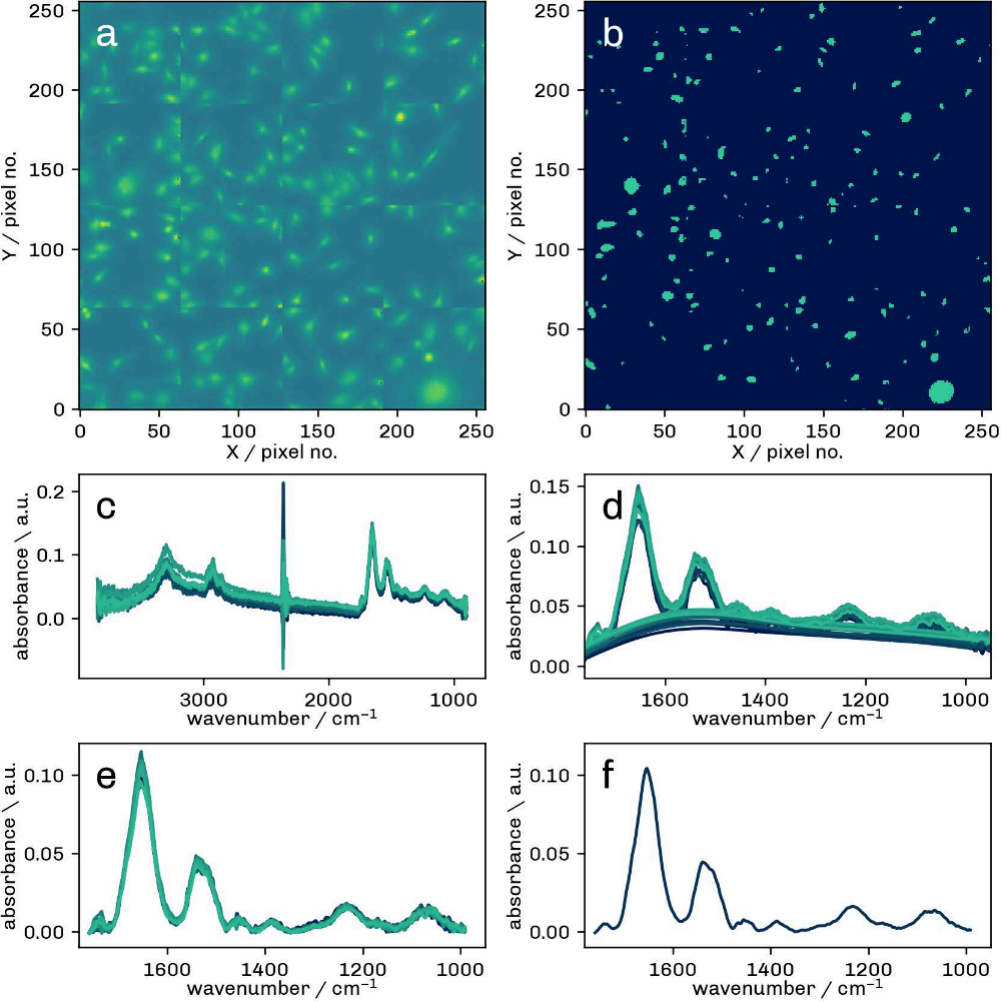
Processing workflow for FTIR hyperspectral images. a) FPA image obtained from the integrated area under the Amide I band (1580 cm^–1^ to 1710 cm^–1^), b) segmentation into ROIs with cell regions (green) and background (dark blue), c) raw spectra, d) baseline correction (BC), e) spectra after BC and EMSC correction, f) resulting average cell spectrum after normalization to the Amide I band.

The remaining 6410 raw spectra (c) were smoothed by a first-order Savitzky–Golay filter with a window length of 7. The smoothing filter settings were adjusted to balance noise suppression and spectral fidelity. Correction for Mie scattering was performed using the Extended Multiplicative Signal Correction (EMSC) package of the openvibspec library.^48^ Mie scattering occurs from (near-)­spherical sample structures and has been shown to severely distort infrared band shapes.^49^ The choice to use EMSC was based on its effectiveness in the removal of Mie scattering artifacts from single-cell measurements.^50^

Next, the baseline was removed (d) using an asymmetric least-squares (AsLS) algorithm^51^ of the pybaseline library.^46^ The AsLS approach is based on asymmetric weights to correct baseline drifts without distorting the spectral bands. It has proven to be most useful for spectra with distinct peaks and smooth baseline drifts,^41^ which is why it was deemed appropriate for this data collection.

After baseline correction, the spectra were trimmed to the wavenumber range of interest (1760 cm^–1^ to 1000 cm^–1^). Due to the use of CaF_2_ coverslips and windows, low wavenumbers below 1000 cm^–1^ are strongly attenuated and cannot be reliably detected. The upper range limit was based on the fact that biological molecules do not absorb in the region of 1800 cm^–1^ to 2800 cm^–1^.^52^ The exact cutoff was set so as to exclude as much noise as possible without truncating the esterified lipid C=O band. Subsequently, the cell mean was calculated from all the measurements collected in the same ROI (e).

In a final step, the spectra were normalized to the Amide I band (f). The Amide I absorption band is representative of the protein backbone. It is a strong, broad and isolated region in the spectrum and can therefore serve as a benchmark for the overall protein content in biological samples.

#### 2.3.2 O-PTIR

In the custom O-PTIR setup, several spectra were recorded as single-point spectra, averaging three spectra per measurement. Measurement locations were chosen systematically for each cell. To begin, the cell nucleus was located via the visible image and the optics were adjusted to obtain the maximum signal. The spectra were then collected at a series of points approximately 500 nm away from the signal maximum in the cardinal directions (up, down, left, right) and, in addition, at a point on the edge of the cells. One sample batch comprising 25–30 cells per class was analyzed with the O-PTIR system. Because the group sizes were considerably smaller than in the collection of FTIR spectra, a standard-deviation approach was not deemed feasible to estimate the noise in the O-PTIR data. As a consequence, all spectra were corrected for the laser chip crossover (see below) and normalized to the Amide I band, and the noise was taken as the mean intensity in the region of 980 cm^–1^ to 1000 cm^–1^, where no O-PTIR signal is present. The SNRs, calculated as the quotient of the class-wise mean intensity at 1650 cm^–1^ and the class-wise mean noise, were 31, 31, 24, and 24 for MDA-MB 231 WT, MDA-MB 231 KO, HUVEC and HAoEC cells, respectively.

The photothermal signal is the difference in the transmitted probe laser intensity with and without the absorption of the pump laser radiation by the sample. The change in the transmitted intensity has the same frequency as the pump laser modulation, while the baseline intensity of the probe laser corresponds to the low-frequency component of the transmission signal. For baseline correction, all raw spectra were therefore divided by the low-frequency part of the transmitted visible laser intensity before further processing.

In a next step, a strong signal artifact resulting from the EC-QCL crossover transition between Chip 3 and Chip 4 had to be removed from the spectra. To this end, the values in the affected spectral region (1265 cm^–1^ to 1355 cm^–1^) were excluded from further analysis. In Figure 2, the missing values were interpolated for better visualization.

**Figure 2:**
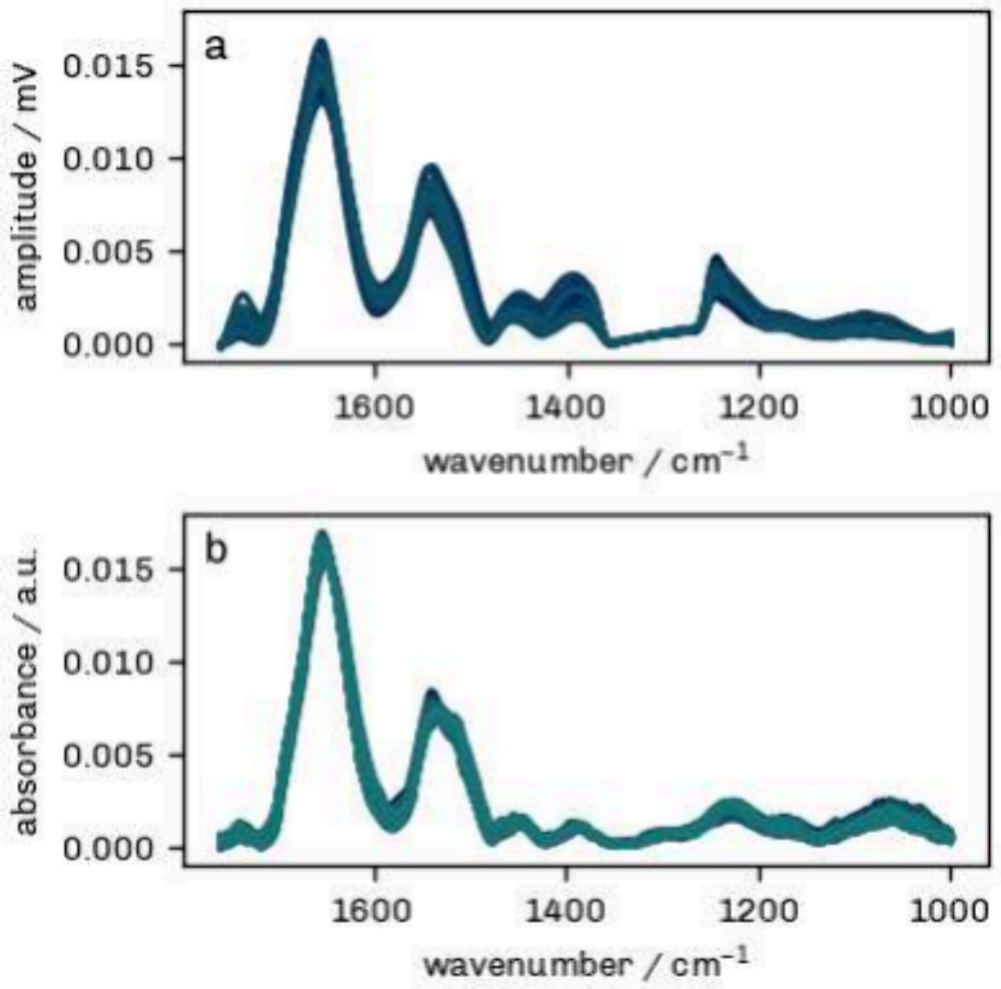
Typical O-PTIR (a) and FTIR (b) spectra after processing. The spectra shown represent all HUVEC cell means included in the classification step. The gap in the O-PTIR spectrum is due to a crossover point in this region.

After calculating average cell spectra, these were smoothed using a first-order Savitzky–Golay filter with a window size of 12 and normalized to the Amide I band. Again, the filter parameters were chosen to preserve the main spectral features but remove unwanted noise. Higher filter orders were tested but did not improve the result. An example of O-PTIR and FTIR spectra after the processing procedure is presented in Figure 2.

### 2.4 Classification and Selection Algorithms

All algorithms were used as implemented in the Python scikit-learn module,^47^ version 1.3.1.

#### 2.4.1 Training and Test Set Split

Each data set was split into a training subset and a test subset considering the weight of each class. In general, for *p* parameters to be fitted in the regression model, it is favorable to use a splitting ratio of $\sqrt{p}\text{:}1$ .^53^ Assuming that only 5% of the available data points are selected by the LASSO subset, the 380 wavenumbers recorded by the FTIR microspectrometer in the range of interest would be reduced to a mere 19 wavenumbers, yielding a training/test set ratio of 4:1. Although this ratio would be higher for the O-PTIR spectra if we considered the larger number of data points (higher spectral resolution), the low number of samples would make cross-validation unreliable. Consequently, the training/test split ratio was set to 80:20 for both the FTIR and O-PTIR data sets. The classification routine was repeated six times with random assignment to training and test sets.

#### 2.4.2 LASSO

The Least Absolute Shrinkage and Selection Operator (LASSO) regression finds a linear least-squares fit to a data set by adding a penalty parameter α. The LASSO algorithm solves the following optimization problem:^54^

$$\underset{m}{\min}\left({∥y-mX∥}_{2}^{2}+\alpha {∥m∥}_{1}\right)$$ 1

where *X* is the covariate matrix, *y* is the response variable, *m* is the coefficient vector, with ∥*m*∥_1_ = |*b*_1_| + |*b*_2_| + ..., and *N* is the number of samples.

The LASSO reduces the number of features on which the solution depends, thereby decreasing the likelihood of overfitting. Using gradient descent approximation to iteratively solve the above optimization problem, it is suitable for variable selection in collinear data sets like spectra.^55^

A series of penalty values α ([Disp-formula eq1]) was input to find the best regression model by cross-validated accuracy score before the final model was computed.

#### 2.4.3 Linear Discriminant Analysis

After the data set had been trimmed to the wavenumber subset selected by the LASSO routine, Linear Discriminant Analysis (LDA) was carried out to build a classifier.

The solver was set to eigenvalue decomposition, which involves the estimation of the covariance matrix. Although matrix calculations are computationally intense, computation time is expected to be reasonable for a reduced number of features like in this case.

Shrinkage regularization was found to boost the performance of the algorithm even for a reduced number of spectral features. Shrinkage improves the generalization of the covariance matrix when the number of training samples is smaller than the number of features.^56^ The optimum shrinkage value was determined via the GridSearchCV module, which brute-forces a range of shrinkage parameters and selects the one with the highest metric.

Each LDA model was cross-validated. Cross-validation was performed with 10 folds for the FTIR data and 3 folds for the much smaller O-PTIR data set.

## 3 Results and Discussion

### 3.1 Spectral Marker Candidates Identified by the LASSO Algorithm

For the FTIR measurements, classifier performance was best for 7–10 wavenumbers (Table S1), whereas 9–13 marker candidates were selected for classification of the O-PTIR data (Table S2) depending on the test run. The number of features within the selected wavenumber subset is related to the LASSO penalty (see section 2.4.2). The slightly larger number of features required for the O-PTIR model can be explained by a higher variation in this data set owing to the higher spectral (1 cm^–1^) and spatial (<700 nm in the lateral direction) resolution of the O-PTIR instrument.^31^ Apart from the more local character of the spectra, the smaller sample size of the O-PTIR data set increases uncertainty in the estimation of parameters.

This having been said, the wavenumbers of the selected spectral features differ slightly between the two data sets but are overall consistent to each other in terms of associated functional groups. The candidate markers identified by LASSO preselection are discussed in detail in the following sections.

#### 3.1.1 Phosphates

The symmetric (ν_s_PO_2_^–^) and asymmetric (ν_as_PO_2_^–^) stretching modes of the phosphate bond^3,57^ are indicators of the DNA and RNA conformation. These spectral regions have been suggested as potential cancer markers^11,26^ and have been a key factor in the classification of breast cancer types into subcategories with varying aggressiveness.^27^

Potential phosphate markers emerged from the analysis of both data sets. In the FTIR data, the algorithm detected C–O stretching of the phosphodiester groups at 1060 cm^–1^ to 1066 cm^–1^,^32^ while ν_as_PO_2_^–^ modes were prominent in the O-PTIR analysis. These were found at 1210 cm^–1^, 1229 cm^–1^ to 1231 cm^–1^, 1236 cm^–1^, and 1242 cm^–1^ to 1243 cm^–1^.

Similar marker bands have been suggested for ovarian cancer cells^11^ and cervical cancer cells.^28^ In ovarian cancer cells, the ν_s_PO_2_^–^ band intensity at 1086 cm^–1^ and the ν_as_PO_2_^–^ intensity at 1242 cm^–1^ differed significantly between various cell lines.^11^ In precancerous cervical cells, a reduced intensity in both ν_s_PO_2_^–^ and ν_as_PO_2_^–^ was found at 1080 cm^–1^ and at 1237 cm^–1^, respectively.^28^

A caveat here is that the cells analyzed in this study were fixed and stored in PBS, which may lead to intracellular phosphate accumulation. Although all cell samples were prepared and stored under the same conditions and by the same laboratory technician, bias due to differences in phosphate uptake or release during sample preparation cannot be fully excluded.

#### 3.1.2 Amide I and II

Proteins are chains of amino acids that are linked by peptide bonds.^58^ The amide bands reflect the secondary structure of proteins, that is, the local folding patterns of this polypeptide chain.^1,32^ The two most common folding patterns are the α helix and the β sheet.^58^

The Amide I vibration absorbs near 1650 cm^–1^ and is dominated by the C=O stretching vibration, with contributions from the CN stretching, the CCN deformation, and the NH bending vibrations.^59^ The conformation of the protein backbone dictates the degree to which the various vibrations contribute to the Amide I mode, making it a sensitive probe of protein secondary structure.^60^

The Amide II mode, centered at 1550 cm^–1^, is mainly a combination of the NH bending vibration and the CN stretching vibration.^59^ Minor contributions come from the CO bending as well as the CC and NC stretching vibrations. The Amide II vibration also reflects secondary structure, but its relationship with the contributing vibrations is more complex than that for the Amide I mode.^61^

Differences in the alpha helical and beta-sheet structures of Amide I have been described between normal and ovarian cancer cells as well as among different types of ovarian cancer cells, suggesting possible structural alterations.^11^ Similarly, a shift of the Amide II band to lower wavenumbers was reported in metastatic cells versus primary melanoma cells.^29^ In abnormal cervical cells, the Amide II band at 1539 cm^–1^ was weaker than in normal cells.^28^

In the FTIR measurements, the LASSO algorithm selected Amide II features at 1552 cm^–1^ to 1558 cm^–1^, 1563 cm^–1^, and 1573 cm^–1^, as well as Amide I features at 1627 and 1629 cm^–1^. The Amide II features at 1541 cm^–1^, 1545 cm^–1^ to 1548 cm^–1^, and 1592 cm^–1^ to 1595 cm^–1^, and the Amide I band at 1645 cm^–1^ to 1648 cm^–1^ emerged as potential markers from the O-PTIR spectra.

#### 3.1.3 CH_2_/CH_3_ Bending

The spectral region of 1450 cm^–1^ to 1465 cm^–1^ is associated with the CH_2_ scissoring and asymmetric CH_3_ bending modes (δ_as_CH_3_) of proteins and lipids.^32,59,62^

The relative intensity of the peak at 1448 cm^–1^ emerged as a prominent feature in several breast cancer cell lines.^30^ In another study scrutinizing the metastatic properties of different cell types, the relative intensity at 1456 cm^–1^ was found to be increased in metastatic versus nonmetastatic colon and melanoma cells, but lower in metastatic MDA-MB-231 cells versus MCF-7 cells.^63^

In the O-PTIR data, a corresponding candidate marker band was detected at 1456 cm^–1^ to 1458 cm^–1^.

#### 3.1.4 Ester C=O Stretching of Phospholipids

The reprogramming of the lipid metabolism has been recognized as a hallmark of cancer and metastasis.^64–66^ In FTIR analysis, the C=O stretching of phospholipids, a main component of the cell membrane,^66^ has been associated with carcinogenesis and migration. In a study aimed at distinguishing between SKBr3 and MCF-7 breast cancer cells, the band ratio of 1650 cm^–1^/1740 cm^–1^ emerged as one of several statistically significant marker candidates.^67^ By the same token, altered band intensities at 1741 cm^–1^ were observed in several cancerous cell lines as compared to normal ovarian cells.^11^ Furthermore, it has been reported that fibroblasts developed a distinct peak at 1721 cm^–1^ after stimulation with MCF-7 breast cancer cells; in addition, the band centered at 1735 cm^–1^ decreased in intensity after cancer stimulation.^68^

Ester C=O vibrational bands were selected in both data sets, at 1743 cm^–1^ for FTIR spectra, and at 1725 cm^–1^ to 1729 cm^–1^ and 1748 cm^–1^ to 1749 cm^–1^ for O-PTIR spectra.

### 3.2 Distribution of Marker Candidates for Different Cell Classes

To establish the relationship of the potential markers identified by the LASSO with cancer and migration properties, the corresponding measurement values were subjected to statistical analysis. As the primary focus of this article is on the novel method of O-PTIR, only the O-PTIR measurements will be discussed in detail. The distribution plots for the FTIR data are provided in the Supporting Information (Figures S2 and S3). Since the selected subset differed slightly for each trial run, a total of 26 wavenumbers emerged from the LASSO regression on the O-PTIR data. Such a variation in selected wavelengths is not unexpected, as the absorption at neighboring wavelengths is often correlated, yielding multiple wavelengths that contain essentially the same information. Some of the marker candidates were therefore merged into groups and replaced by the group mean.

Histograms were plotted by cell class and potential marker band to visualize differences in signal intensities. As evident from Figures 4 and 5, some values are not normally distributed. Hence, a Mann–Whitney *U* test was performed for each selected marker band to quantify the differences between cell classes (Table 1, Figure 3).

**Table 1:** Mann–Whitney *U* Statistics and *p* Values per Cell Group and Selected Band for the O-PTIR Data Set^a^

**Band [cm** ^**–1**^ **]**	**Cancer**	**Healthy**	***p***	**WT**	**KO**	***p***
1210	1311	491	0.00037	175	521	0.00205
1229–1231	1061	741	0.16529	170	526	0.00152
1236	1004	798	0.37258	163	533	0.00098
1242–1243	943	859	0.71809	156	540	0.00062
1456–1458	1359	443	6.9e-05	403	293	0.33014
1541	773	1029	0.26737	378	318	0.59811
1545–1548	702	1100	0.08421	396	300	0.39602
1592–1595	935	867	0.77073	232	464	0.03904
1645–1648	726	1076	0.12902	265	431	0.14044
1725–1729	1051	751	0.19343	464	232	0.03904
1748–1749	1517	285	8.6e-08	304	392	0.43699

a Tests were made between Cancer (MDA-MB 231 WT + MDA-MB 231 KO) vs. Healthy (HUVEC + HAoEC) and MDA-MB 231 WT vs. MDA-MB 231 KO cells. Groups are considered different when *p* < 0.05.

**Figure 3:**
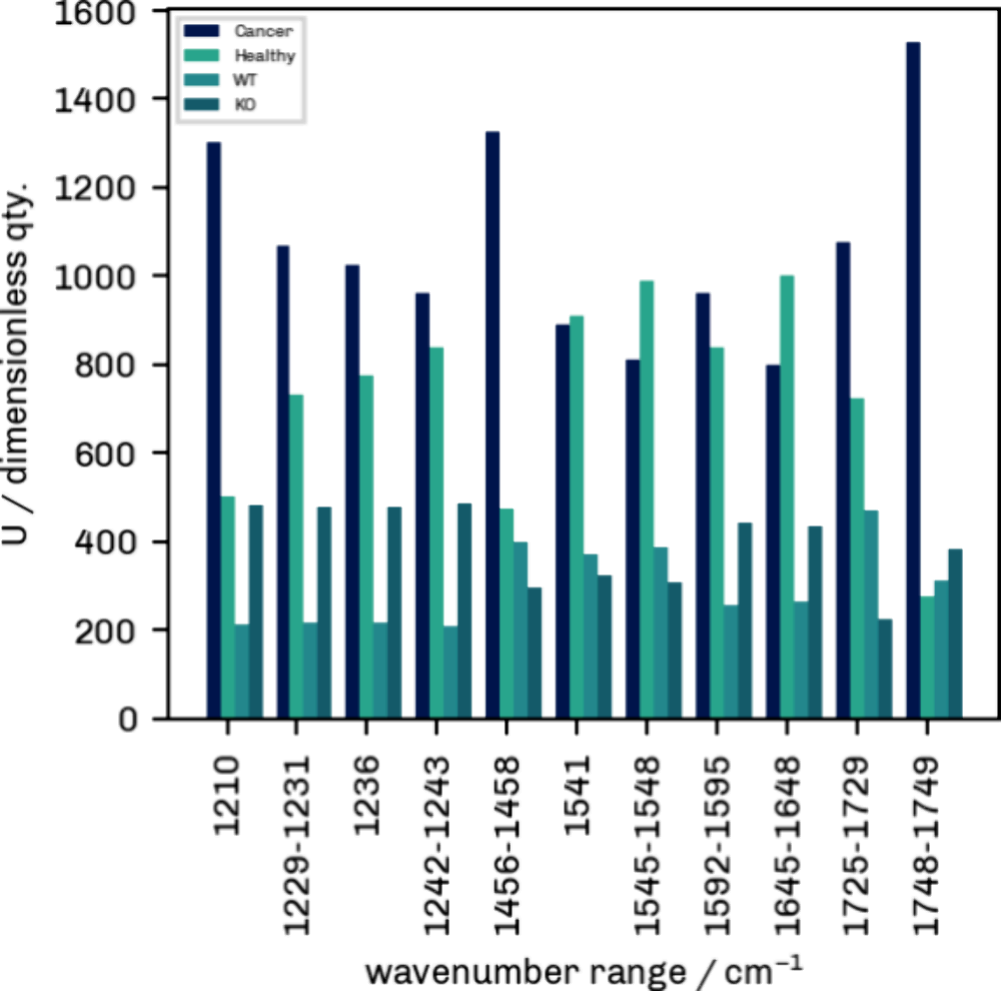
Mann–Whitney *U* test statistics for Cancer/Healthy and MDA-MB 231 WT/MDA-MB 231 KO groups for each IR marker band.

#### 3.2.1 Cancer Cells versus Endothelial Cells

In a first analysis, the MDA-MB 231 WT and MDA-MB 231 KO cells were combined to form a “Cancer” cell class. Figure 4 shows the distribution of signal values for healthy endothelial cells versus cancer cells. The *y* axis indicates the number of samples, and the *x* axis represents the intensity, which was grouped into 12 bins.

**Figure 4:**
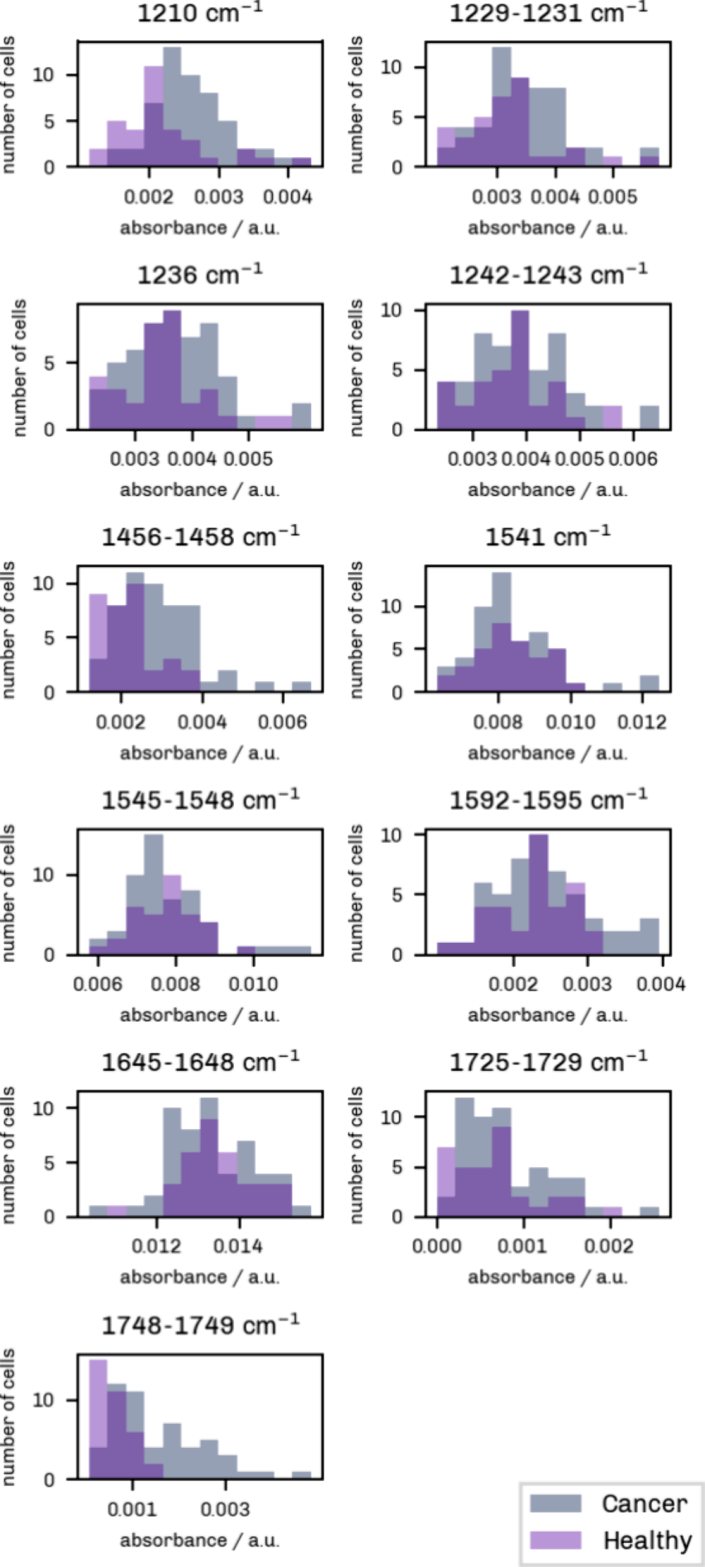
Histograms showing the distribution of O-PTIR signal intensities for “Cancer” (MDA-MB 231 WT + MDA-MB 231 KO, gray) and “Healthy” (HUVEC+HAoEC, light purple) cell types. Twelve bins were used here. The dark purple areas mark the overlap between the two groups.

Overall, there is a strong overlap between the two cell classes as illustrated by the dark purple areas. For the potential phosphate markers at 1210 cm^–1^, 1229 cm^–1^ to 1231 cm^–1^, and 1236 cm^–1^, the signal is generally higher in the cancer samples. This is in line with the results disclosed by Amjad et al.,^1^ where a higher band intensity at 1209 cm^–1^ and a lower intensity at 1238 cm^–1^ were reported. However, only the measurement at 1210 cm^–1^ turned out to be significant (Mann–Whitney *U* test, *p* < 0.01).

A similar trend can be observed for the CH_2_/CH_3_ band at 1456 cm^–1^ to 1457 cm^–1^ as well as the esterified lipid bands at 1725 cm^–1^ to 1729 cm^–1^ and 1748 cm^–1^ to 1749 cm^–1^. The CH_2_/CH_3_ band and the 1748 cm^–1^ to 1749 cm^–1^ band resulted significant (*p* < 0.01). A change in the band around 1740 cm^–1^ was also described by Li et al.^11^ in their FTIR analysis of ovarian cancer cells.

In the Amide-II bands at 1541 cm^–1^ and 1545 cm^–1^ to 1548 cm^–1^, the distribution of values is shifted to lower intensities for the cancer measurements. This is also true for the marker region detected in the Amide-I band (1645 cm^–1^ to 1648 cm^–1^). Although not statistically significant, these trends corroborate the results reported in similar studies.^1,28^

#### 3.2.2 MDA-MB 231 WT versus MDA-MB 231 KO Cells

To analyze the invasive properties, a comparison was made between the MDA-MB 231 WT and MDA-MB 231 KO specimens (Figure 5). A slightly lower number of 10 intensity bins was chosen because of the lower number of samples in the two cancer groups alone (as opposed to the entire data set).

**Figure 5:**
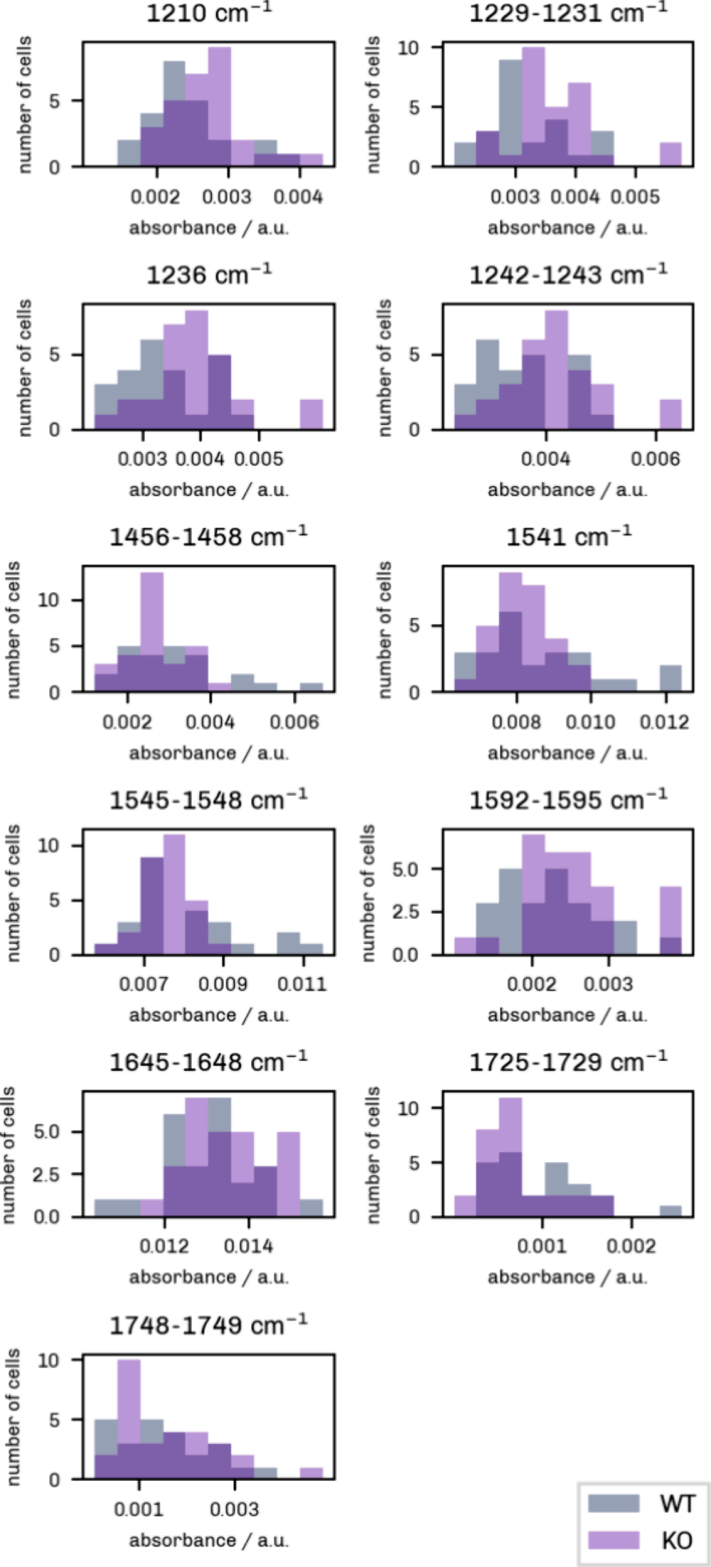
Histograms showing the distribution of O-PTIR signal intensities for MDA-MB 231 WT (“WT”, gray) and MDA-MB 231 KO (“KO”, light purple) cell types. Ten bins were used. The dark purple areas mark the overlap between the two groups.

In the ν_as_PO_2_^–^ region, the intensity at the bands of 1210 cm^–1^, 1229 cm^–1^ to 1231 cm^–1^, 1236 cm^–1^, and 1242 cm^–1^ to 1243 cm^–1^ was higher in the KO samples. Indeed, the Mann–Whitney *U* test revealed a significant difference (*p* < 0.05) between the MDA-MB 231 WT and the MDA-MB 231 KO samples in all the detected phosphate marker candidates. Two other bands resulted significant (*p* < 0.05): the band at 1592 cm^–1^ to 1595 cm^–1^, which was lower for highly invasive cells, and the potential marker at 1725 cm^–1^ to 1729 cm^–1^, which was higher in the MDA-MB 231 WT group.

### 3.3 Classification by LDA

After the data set had been reduced to the wavenumbers selected by the LASSO routine, the remaining data were randomly allocated to a training and test set, and a classification model was built using LDA. Randomization and classification were repeated 6 times.

Figures 6 and 7 display the confusion matrices for the FTIR and O-PTIR data set, respectively. The percentages are mean values obtained from the 6 classification runs as described above. Diagonal and off-diagonal elements correspond to the percentages of correct and incorrect predictions.

**Figure 6:**
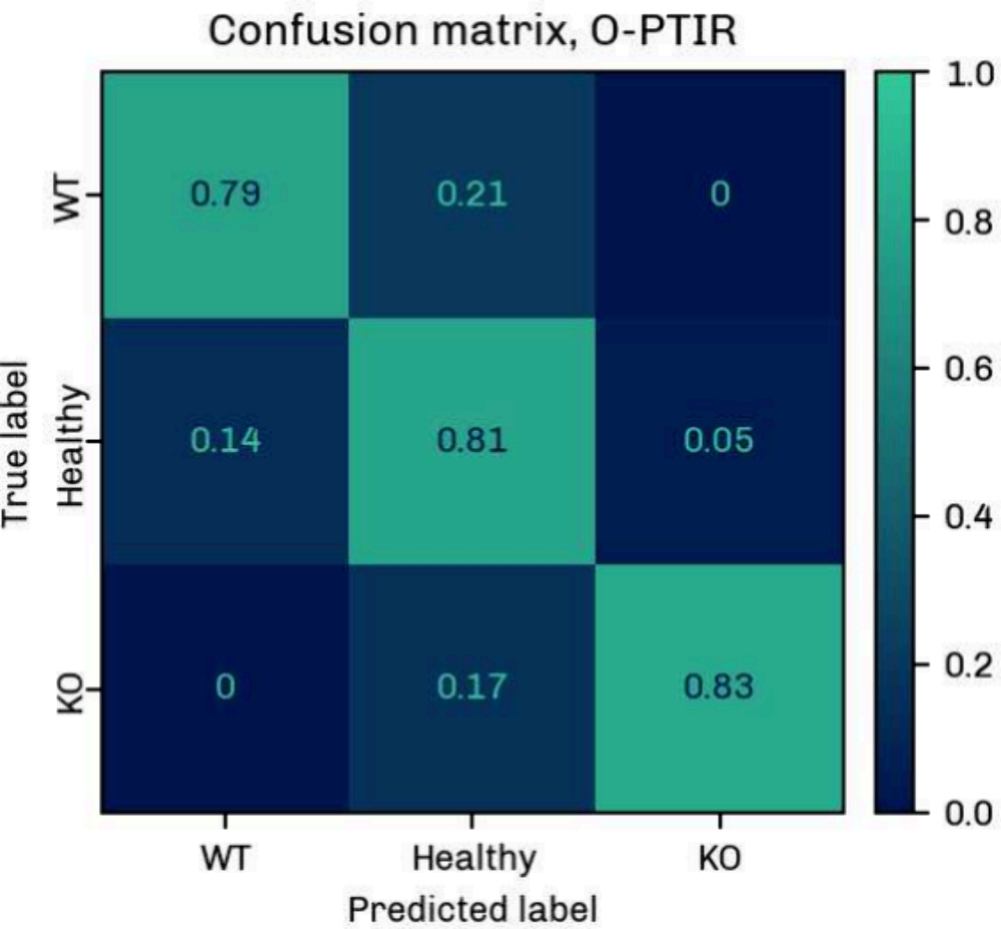
Confusion matrix for the classification of MDA-MB 231 WT, MDA-MB 231 KO and Healthy cell classes using the O-PTIR data. The matrix shows the mean classification values over 6 runs, with diagonal elements representing correct and off-diagonal elements representing incorrect predictions.

**Figure 7:**
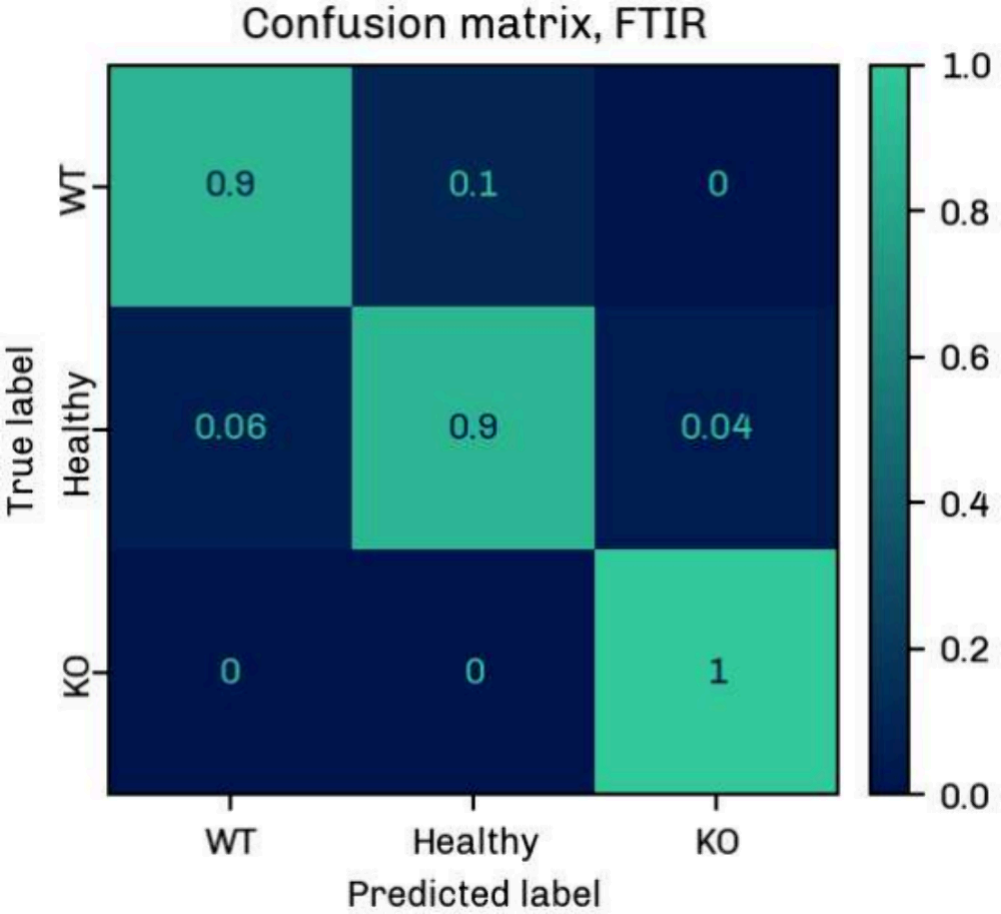
Confusion matrix for the classification of MDA-MB 231 WT, MDA-MB 231 KO and Healthy cell classes using the FTIR data. The matrix shows the mean classification values over 6 runs, with diagonal elements representing correct and off-diagonal elements representing incorrect predictions.

The mean classification accuracy (Table S4) was 93 ± 3% for the large FTIR data set; the weighted F1 score, sensitivity, and specificity were 93 ± 2%, 93 ± 2%, and 96 ± 1% (Tables S5, S7, and S8). It should be noted here that the classification accuracy may be influenced by interbatch differences, which were not assessed in this study due to a lack of data and considerable differences in batch size.

In the O-PTIR measurements, the classes were identified with a mean accuracy of 81 ± 6% (Table S4), a weighted F1 score of 81 ± 6%, a weighted sensitivity of 81 ± 6%, and a weighted specificity of 90 ± 4% (Tables S6–S8). The accuracies were verified by cross-validation (Tables S9 and S10).

There is some confusion between the cell lines, which is somewhat expected given the small chemical difference between the MDA-MB 231 WT and MDA-MB 231 KO groups. The higher spatial and spectral resolution of the O-PTIR technique and the small number of samples reduce the overall classification accuracy for the O-PTIR data. The small sample number is also reflected by the higher uncertainty of 6%. In addition, the mean cell spectra comprised only 5–6 point spectra, which may lead to greater variability in the O-PTIR data. In view of the above, these classification results are preliminary and are expected to change with a larger pool of analyzed cells, the number of points averaged per cell, and the number of batches included in the analysis.

Drawing direct comparisons to immunohistochemical research is challenging at best. There is a high degree of heterogeneity in cell and tissue types, which greatly influences the performance of antibody markers. As the sensitivity and specificity of antibodies vary greatly, cancer detection often requires a combination of markers that is tailored to a specific tumor or cell type, making it impossible to pinpoint standards for sensitivity and specificity that are valid across cells and tissues of different origin. As an example for research on triple-negative breast cancers, Thike et al.^69^ were able to differentiate basal-like cancers within triple-negative cancers using a combination of three markers with a specificity of 100% and a sensitivity of 78%. With regard to individual markers, the sensitivity and specificity in identifying primary breast carcinoma have been reported to be 93% and 92% for mammaglobin, and 85% and 99% for GCDFP-15.^70^

## 4 Concluding Summary

Our findings confirm that the spectra recorded with the home-built O-PTIR instrument exhibit the same qualitative features as spectra obtained with a commercial FTIR microspectrometer.

The potential marker bands selected by the LASSO routine as distinctive features for the highly invasive MDA-MB 231 WT cells, noninvasive MDA-MB 231-KO cells and the healthy controls (HUVEC and HAoEC) include the Amide I and Amide II, phosphate, CH_2_/CH_3_ bending and esterified lipid spectral regions. A larger number of variables was needed to establish a classification model based on the O-PTIR measurements, which reflects the higher spatial and spectral resolution of the technique. Despite the higher variability in the O-PTIR as compared to the FTIR spectra, the candidate markers output by the LASSO are consistent for both data sets.

In-depth analysis of the O-PTIR marker candidates showed differences between the cancerous (MDA-MB 231 WT + MDA-MB 231 KO) and the healthy populations as well as between the MDA-MB 231 WT and MDA-MB 231 KO cell lines, which only differed in the presence or absence of the JAG1 notch ligand. Our findings suggest vibrational bands distinguishing cancer and healthy samples in the ν_as_PO_2_^–^, CH_2_/CH_3_ bending and esterified lipid regions. For the MDA-MB 231 WT and MDA-MB 231 KO groups, significant differences were found for all LASSO-selected ν_as_PO_2_^–^ bands, in the Amide II region and in the esterified lipid region. These results are in line with existing evidence implicating phosphate and esterified lipid vibrational groups as potential markers in the study of cancer and migratory properties at the cellular level.^11,26,27^

Subsequent classification by LDA yielded a mean sensitivity and specificity of 93 ± 2% and 96 ± 1% for the FTIR versus 81 ± 6% and 90 ± 4% for the O-PTIR spectra. The reason for the lower performance of the algorithm on the O-PTIR data set lies in the higher spatial and spectral resolution of the O-PTIR instrument and the smaller group sizes. O-PTIR cell measurements comprised a smaller number of spectra per cell, which may not be enough to even out local chemical heterogeneities in biological samples. These classification results are expected to improve with a larger number of measurements and cells. Furthermore, this study does not address the influence of interbatch effects and a larger number of measurements per cell on the classification accuracy. More research is needed to improve the generalization of these preliminary results beyond the current setup and translate them into clinical applicability.

Notwithstanding these limitations, the fact that the algorithm can identify each class in both data sets (even with lower accuracy) substantiates the general validity of the approach taken and underscores the potential of the O-PTIR technique as a tool for the detection of high-risk invasive cells.

These results confirm the capabilities of our transmission-mode O-PTIR prototype and have established a baseline for future research. Building on the experience gained in this work, further efforts will be dedicated to the study of biological samples in aqueous environments and on-chip systems. This will provide further insights into the applicability of O-PTIR spectro-microscopy to the study of hydrated tissues and live cells in aqueous media.

## Supplementary Material



## Data Availability

The data underlying this study are openly available in Zenodo at DOI: 10.5281/zenodo.15774784.

## Notes

Sketch of the setup, wavenumbers identified by the LASSO routine for the FTIR and O-PTIR data sets, and histograms showing the distribution of FTIR signal intensities for different cell classes (PDF)
