# Supplementary material for: Investigating Spectral Biomarker Candidates for Migratory Potential in Cancer Cells Using Micro-FTIR and O‑PTIR Spectroscopy
Source: ACS Meas Sci Au. 2026 Jan 21;6(1):96–106. doi: 10.1021/acsmeasuresciau.5c00132 (PMC12921600; doi:10.1021/acsmeasuresciau.5c00132)
Supplement: Supplementary file 1 [file tg5c00132_si_001.pdf]

Supporting Information: Investigating Spectral Biomarker Candidates for Migratory Potential in Cancer Cells Using Micro-FTIR and O-PTIR Spectroscopy

Elisabeth Holub,† Nikolaus Hondl,† Kai-Lan Lin,‡ Marjaana Parikainen,‡ Cecilia Sahlgren,‡,¶,§,‖ Bernhard Lendl,⊥ and Georg Ramer*,†,#

†*Institute of Chemical Technologies and Analytics, TU Wien, 1060, Wien, Austria*

‡*Faculty of Science and Engineering, Åbo Academi, 20500, Turku, Finland*

¶*InFLAMES Research Flagship, Åbo Akademi University and University of Turku, 20500, Turku, Finland*

§*Department of Biomedical Engineering, Eindhoven University of Technology, 5631 BN, Eindhoven, the Netherlands*

‖*Institute for Complex Molecular Systems (ICMS), Eindhoven University of Technology, 5612 AJ, Eindhoven, the Netherlands*

⊥*Institute of Chemical Technologies and Analytics, TU Wien, 1060 Wien, Austria*

#*Christian Doppler Laboratory for Advanced Mid-Infrared Laser Spectroscopy in (Bio-)process Analytics, TU Wien, 1060, Wien, Austria*

E-mail: georg.ramer@tuwien.ac.at

1 Spectral ranges of MIRcat-QCT-z

Table S1: Spectral ranges of the MIRcat-QCT-z

	Chip 1	Chip 2	Chip 3	Chip 4
Range / cm^{-1}	2936-2347	1797-1347	1505–1195	1271–930

2 Wavenumbers Selected by the LASSO Routine

Table S2: Wavenumbers of interest in cm^{-1}identified by the LASSO operator for the FTIR data.

**Test no.**	**$\nu_s PO_2^-$**			**Amide II**						**Amide I**	**Lipid esters**
	1060	1062	1066	1552	1554	1556	1558	1563	1573-1575	1627-1629	1743
1	•	•	•			•		•	•	•	•
2		•	•	•		•	•	•		•	•
3	•	•	•	•		•		•	•	•	•
4	•	•	•		•	•		•		•	•
5		•	•			•		•	•	•	•
6	•	•	•	•		•	•		•		•

Table S3: Wavenumbers of interest identified by the LASSO operator for the O-PTIR data.

**Test no**	**$\nu_{as} PO_2^-$**				**CH bending**	**Amide II**			**Amide I**	**Lipid esters**	
	1210	1229-1231	1236	1242-1243	1456-1458	1541	1545-1548	1592-1595	1645-1648	1725-1729	1748-1749
1			•		•	•		•	•	•	•
2	•	•			•		•	•	•	•	•
3			•	•	•			•	•	•	•
4		•		•	•		•	•	•	•	•
5		•			•		•	•	•	•	•
6		•	•		•		•	•	•	•	•

3 Custom O-PTIR Setup

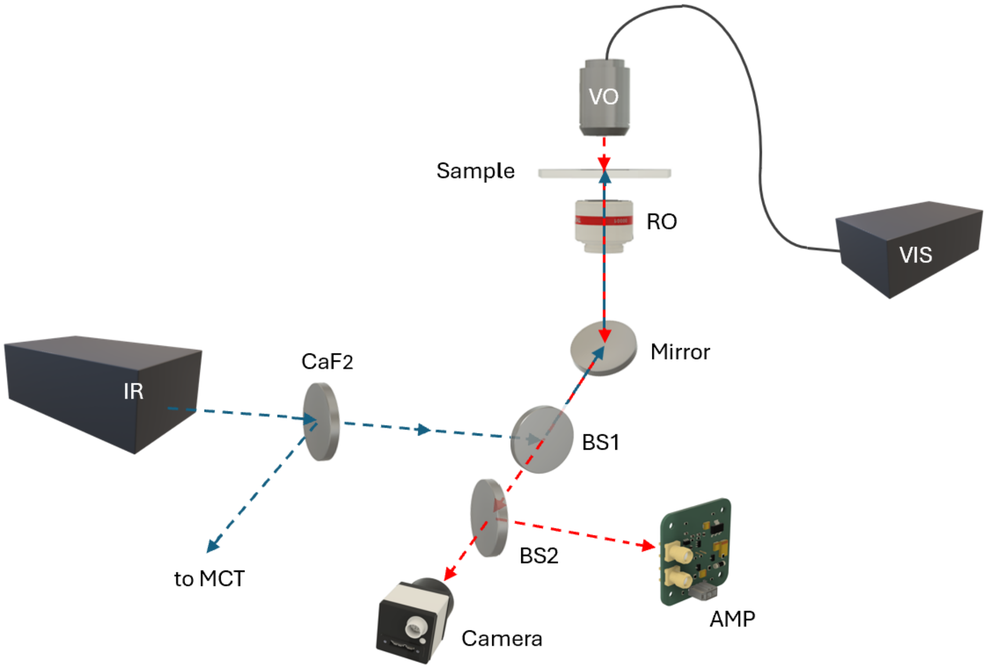

Figure S1: Custom O-PTIR instrument using a tunable IR laser (blue beam path) and a continuous 633 nm visible (VIS) laser (red beam path). IR: infrared laser; VIS: visible laser; VO: visible-light objective; RO: reflective objective; CaF_2: CaF_2 wedged window; AMP: pre-amplifier; BS: beam splitter.

4 Distribution of Markers for FTIR Measurements

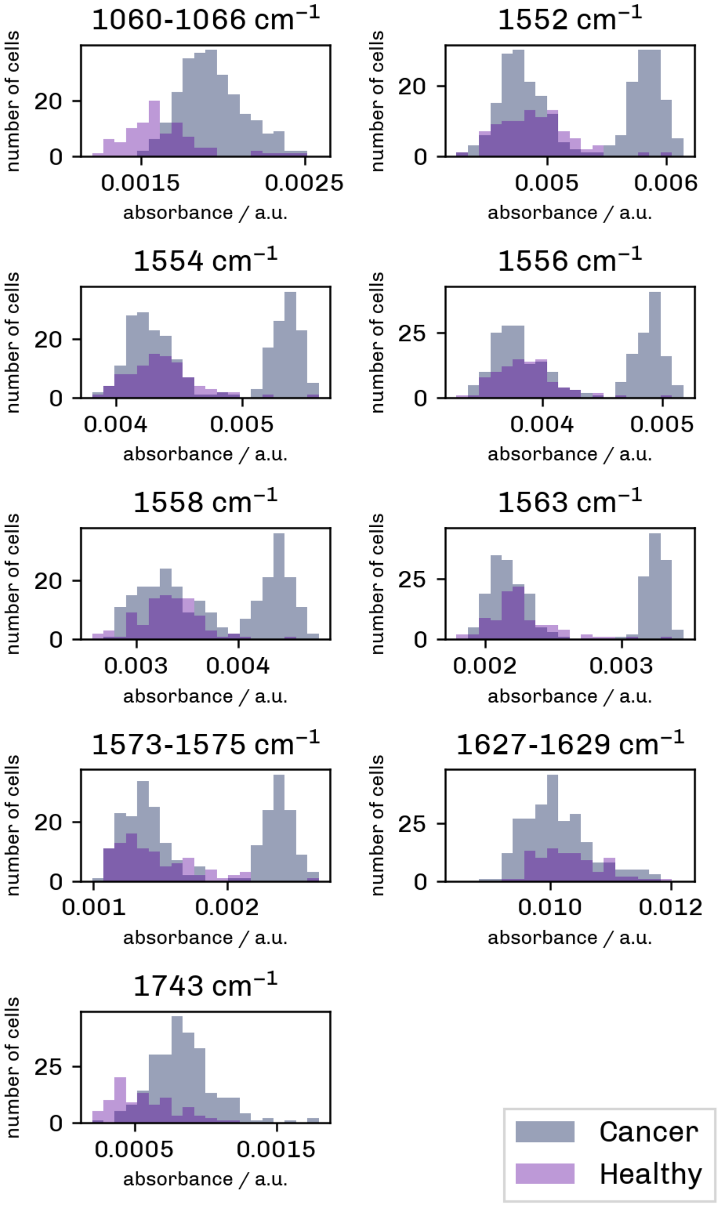

Figure S2: Histograms (20 bins) showing the distribution of FTIR signal intensities for MDA-MB 231 ("Cancer", grey) and HUVEC+HAoEC ("Healthy", light purple) cell types. The dark purple areas mark the overlap between the two groups.

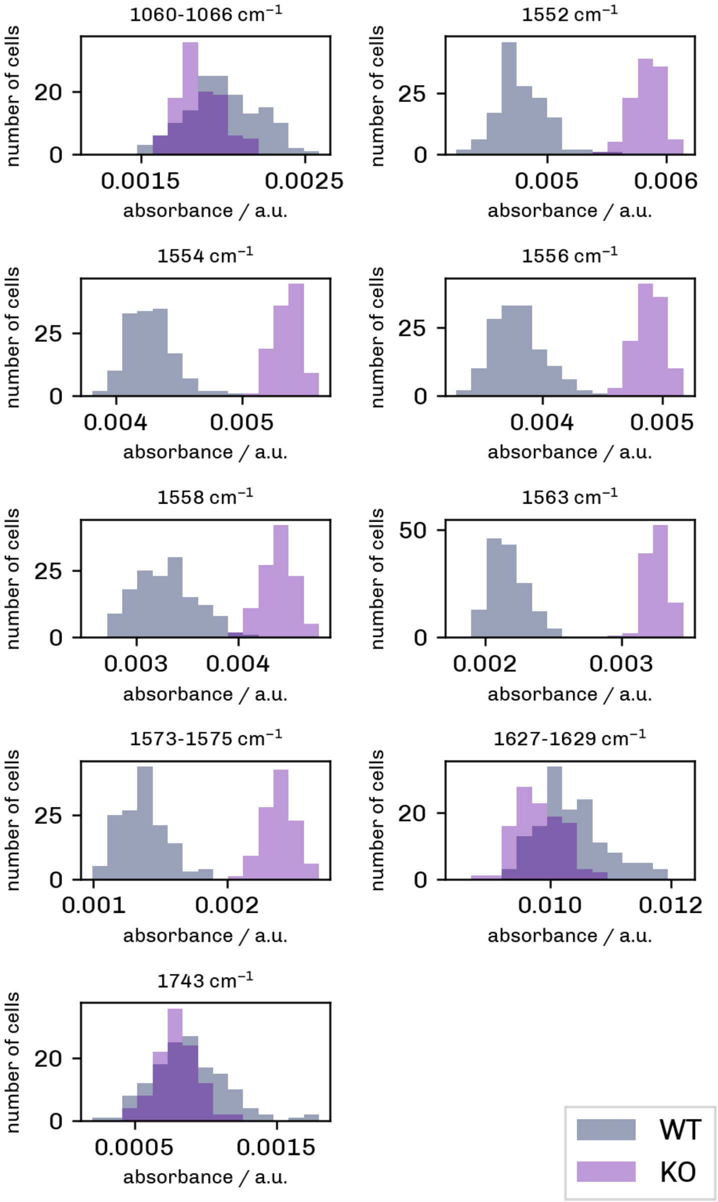

Figure S3: Histograms (15 bins) showing the distribution of FTIR signal intensities for WT (grey) and KO (light purple) MDA-MB 231 cell types.The dark purple areas mark the overlap between the two groups.

5 Classification Accuracy, Sensitivity and Specificity

Table S4: Classification accuracy (6 test runs) for the FTIR and O-PTIR data sets.

	**Test 1**	**Test 2**	**Test 3**	**Test 4**	**Test 5**	**Test 6**	**Mean ± Std**
**FTIR**	0.9701	0.9403	0.9403	0.9254	0.9104	0.9104	0.93 ± 0.02
**O-PTIR**	0.8000	0.8000	0.8000	0.8000	0.9333	0.7333	0.81 ± 0.06

Table S5: Weighted F1 values for the FTIR data.

	**F1**	**Class Size**	**Weighted F1**	**Mean acc.**
**WT**	0.92	143		
**KO**	0.99	110	0.93	0.93 ± 0.02
**OK**	0.87	93		

Table S6: Weighted F1 values for the O-PTIR data.

	**F1**	**Class Size**	**Weighted F1**	**Mean acc.**
**WT**	0.79	24		
**KO**	0.87	29	0.81	0.81 ± 0.06
**OK**	0.78	34		

Table S7: Sensitivity for the FTIR and O-PTIR data sets.

	**Test 1**	**Test 2**	**Test 3**	**Test 4**	**Test 5**	**Test 6**	**Mean ± Std**
**FTIR**	0.9	0.94	0.93	0.96	0.9	0.93	0.93 ± 0.02
**O-PTIR**	0.79	0.81	0.74	0.81	0.79	0.93	0.81 ± 0.06

Table S8: Specificity for the FTIR and O-PTIR data sets.

	Test 1	Test 2	Test 3	Test 4	Test 5	Test 6	Mean ± Std
**FTIR**	0.94	0.97	0.97	0.98	0.95	0.97	0.96 ± 0.01
**O-PTIR**	0.87	0.91	0.86	0.9	0.88	0.97	0.90 ± 0.04

6 Cross-Validation

Table S9: 10-fold cross validation for the FTIR data including the mean and standard deviation (Std) of every test run.

	Fold 1	Fold 2	Fold 3	Fold 4	Fold 5	Fold 6	Fold 7	Fold 8	Fold 9	Fold 10	Mean	Std
Test 1	0.943	0.886	0.914	0.943	0.971	0.943	0.912	0.971	0.941	0.912	0.9336	0.0273
Test 2	0.943	0.886	0.943	0.943	0.971	0.943	0.941	0.971	0.941	0.912	0.9394	0.0222
Test 3	0.943	0.886	0.943	0.943	0.971	0.943	0.941	0.971	0.941	0.912	0.9394	0.0222
Test 4	0.943	0.886	0.943	0.943	0.971	0.943	0.941	0.971	0.941	0.912	0.9394	0.0222
Test 5	0.943	0.886	0.914	0.971	0.943	0.943	0.912	0.971	0.941	0.941	0.9365	0.0244
Test 6	0.943	0.886	0.914	0.971	0.943	0.943	0.912	0.971	0.941	0.941	0.9365	0.0244

Table S10: 3-fold cross validation for the O-PTIR data including the mean and standard deviation (Std) of every test run.

	Fold1	Fold2	Fold3	Mean	Std
Test 1	0.793	0.862	0.759	0.805	0.044
Test 2	0.862	0.759	0.793	0.805	0.042
Test 3	0.793	0.793	0.793	0.793	0.000
Test 4	0.862	0.862	0.759	0.828	0.060
Test 5	0.828	0.793	0.759	0.793	0.035
Test 6	0.793	0.724	0.759	0.759	0.035
